# Δ10(E)-Sphingolipid Desaturase Involved in Fusaruside Mycosynthesis and Stress Adaptation in *Fusarium graminearum*

**DOI:** 10.1038/srep10486

**Published:** 2015-05-21

**Authors:** Yuan Tian, Guo Y. Zhao, Wei Fang, Qiang Xu, Ren X. Tan

**Affiliations:** 1 Institute of Functional Biomolecules, State Key Laboratory of Pharmaceutical Biotechnology, Nanjing University, Nanjing 210093, P. R. China

## Abstract

Sphingolipids are biologically important and structurally distinct cell membrane components. Fusaruside (1) is a 10,11-unsaturated immunosuppressive fungal sphingolipid with medical potentials for treating liver injury and colitis, but its poor natural abundance bottlenecks its druggability. Here, fusaruside is clarified biosynthetically, and its efficacy-related 10,11-double bond can be generated under the regioselective catalysis of an unprecedented Δ10(E)-sphingolipid desaturase (Δ10(E)-SD). Δ10(E)-SD shares 17.7% amino acid sequence similarity with a C9-unmethylated Δ10-sphingolipid desaturase derived from a marine diatom, and 55.7% with Δ8(E)-SD from *Fusarium graminearum*. Heterologous expression of Δ10(E)-SD in *Pichia pastoris* has been established to facilitate a reliable generation of 1 through the Δ10(E)-SD catalyzed desaturation of cerebroside B (2), an abundant fungal sphingolipid. Site directed mutageneses show that the conserved histidines of Δ10(E)-SD are essential for the 10,11-desaturation catalysis, which is also preconditioned by the C9-methylation of the substrate. Moreover, Δ10(E)-SD confers improved survival and faster growth to fungal strains at low temperature and high salinity, in parallel with to higher contents of 1 in the mycelia. Collectively, the investigation describes a new Δ10(E)-sphingolipid desaturase with its heterologous expression fundamentalizing a biotechnological supply of 1, and eases the follow-up clarification of the immunosuppression and stress-tolerance mechanism.

Sphingolipids are a diverse class of lipids that play important biological roles as structural cell membrane components and cell signaling molecules in eukaryotic cells. Sphingolipids biosynthetically derive from sphingosine that is a long chain amino alcohol acylated with a long chain fatty acid to form ceramide as a “core” of sphingolipids^1^. Because of their functions in cytokinesis^2^ and intercellular signaling^3^, sphingolipids are useful or potential therapeutic targets for managing infections^4^,^5^, cancers^6^, Alzheimer’s disease and asthma^7^, diabetes mellitus^8^ and other metabolic disorders in human^9^, as well as abiotic and biotic stresses in plants^10^. Fusaruside (**1**) is an immunosuppressive sphingolipid, first characterized as a minor compound from *Fusarium semitectum* IFB-121 (initially identified as *Fusarium* sp. IFB-121) residing in *Quercus variabilis* barks^11^, and later re-isolated again as a low abundance sphingolipid from *F. oxysporum* associated with *Cinnamomum kanehirae* barks^12^. Fusaruside is structurally unique in carrying a 9-methyl-4,8,10-sphingatrienine chain (trienic LCB), which is thought to contribute to its efficacy in treating T-cell-mediated liver injury and colitis via regulating STAT1 signaling^13^,^14^. However, the druggability of **1** remains bottlenecked by its unsolved supply issue although it can be synthesized using inevitably some toxic agents^15^. Therefore, the production of **1** is highly desired to be engineered on a biotechnological basis.

Pattern analysis of fungal sphingolipid structures underscores that dienic LCB (9-methyl-4,8-sphingadienine) is quite common^1^. The sphingosine branch pattern of sphingolipids seems kingdom-dependent, and the fungal sphingolipids usually possess a C9-methyl group in their main aliphatic chains, which is absent in the characterized plant cerebrosides^1^. However, the trienic LCB possessed by **1** is extremely rare. More surprisingly, compound **1** is the sole terrestrial organism-derived sphingolipid with such a C9-methylated trienic LCB motif, and it is produced in trace quantity by two *Fusarium* endophytes residing in the perennial trees *Quercus variabilis* and *Cinnamomum kanehirae*, which are repeatedly exposed to the cold winter climate^11^,^12^. Other trienic LCB based sphingolipids have been only detected in a very small number of marine creatures including protist *Thraustochytrium globosum*^1^, anemone *Metridium senile*^1^, sponge *Agelas mauritianus*^1^, ascidian *Phallusia fumigate*^1^, and starfishes *Ophidiaster ophidiamus*^1^, *Narcissia canariensis*^16^, *Cosmasterias lurida*^17^ and *Asterias amurensis*^18^. Moreover, the marine diatom *Thalassiosira pseudonana* contains a sphingolipid Δ10-desaturase that enables the 10,11-desaturation of the C9-unmethylated sphingosine chain^19^. We therefore hypothesized that the knowledge about fusaruside biosynthesis might be of particular significance, and further presumed that the fungal tolerance to cold and high salinity could be conferred by its production of cell membrane sphingolipids with the trienic LCB motif. Inspired by the observation, this work was performed to clarify the biosynthetic pathway of **1**, to identify, clone and overexpress the novel enzyme, Δ10(E)-sphingolipid desaturase (Δ10(E)-SD) that catalyzes the key step of the fusaruside biosynthetic pathway in *Fusarium* species (Fig. 1). Moreover, Δ10(E)-SD confers upon fungal species the ability to survive in extreme environments characterized by low temperature and high salinity.

## Results

### Identification of a new desaturase from fusaruside-producing fungal strains

To overcome the genetic obscurity of *F. semitectum* IFB-121 (original fusaruside producer), we decided to screen for an alternative fusaruside generator from fully sequenced *Fusarium* strains *F. graminearum* CBS123657, *F. oxysporum* CBS123668 and *F. verticillioides* CBS123670. Thus, the sphingolipid-enriched fractions derived from these fungal cultures were analyzed by liquid chromatography hyphened with mass spectrometry (LC-MS). *F. graminearum* was evidenced to produce **1** (minor) and **2** (major) from the Na^+^-liganded molecular ions at *m/z* 774.54907 and 776.56470, respectively (Fig. 2A,B). Reminiscent of the difference between **1** and **2** in chemical structure and abundance, we postulated that the biosynthesis of **1** might involve the 10,11-desaturation of **2**. However, the sphingolipid 10,11-desaturase in fungi remains unknown although some enzymes catalyzing other desaturations of sphingolipids have been addressed^19^,^20^. Using a combined bioinformatic/genetic approach, a scheme was devised to search for a *Fusarium* gene encoding the 10,11-desaturase capable of generating the 10,11-double bond of the trienic LCB, a key step in the **1** biosynthesis.

**Figure 1:**
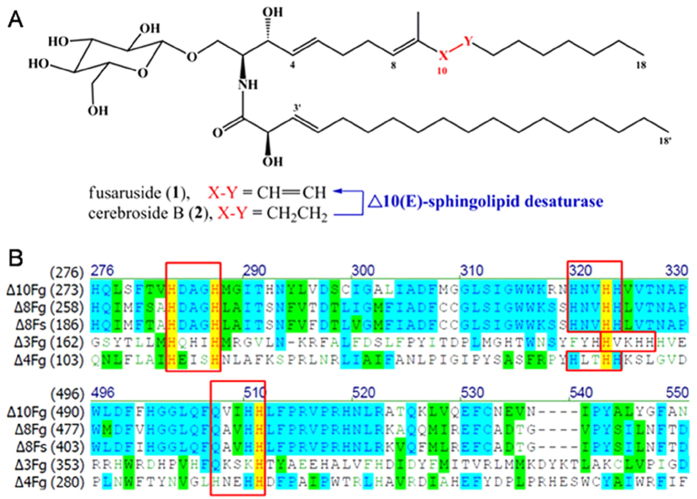
Δ10(E)-sphingolipid desaturase (Δ10(E)-SD) as a new regiospecific biocatalyst. (**A**) Δ10(E)-SD generates **1** from **2** in a regiospecific manner. (**B**) Amino acid sequence alignment of sphingolipid desaturases including Δ8(E)- (Δ8Fg, GenBank: XP381893), Δ4(E)- (Δ4Fg, GenBank: XP390550) and N-Acyl-Δ3(E)-SD (Δ3Fg, GenBank: ACJ35480). Δ10(E)-SD shares only 55.7%, 47.5%, 19.3% and 20.8% similarity (in the consensus sequence region) with those ORFs, respectively. Fg: *Fusarium graminearum*, Fs: *F. semitectum*. Red box surrounds conserved histidine cluster motifs (HX(3or4)H, HX(2or3)HH, and Q/HX(2or3)HH), characteristic structural features of desaturases.

Using genome data and *Fusarium* comparative database (http://www.broad.mit.edu/annotation/fungi/fusarium/), a candidate open reading frame (ORF) homologous to relevant fatty acid desaturases [HX_(3 or 4)_H]X_(20–50)_ [HX_(2 or 3)_HH] X_(100–200)_ [Q/HX_(2 or 3)_HH] was identified. When the ORF corresponding to *F. semitectum* Δ8(E)-SD (GenBank: KC787353) was used as a query, the search identified FGSG_09845, containing an unknown ORF of 1,725 bp (574 amino acids (aa))in the genome of *F. graminearum*. ClustalX analysis^21^ showed that FGSG_09845 shares only 55.7% amino acid sequence identity with the most similar desaturases characterized previously, including Δ4(E)- (GenBank: XP390550), Δ8(E)- (GenBank: XP 381893), N-Acyl-Δ3(E)-SDs (GenBank: ACJ35480) from *F. graminearum*, and Δ8(E)-SD (GenBank: KC787353) from *F. semitectum* (Fig. 1B). The FGSG_09845 ORF had less than 20% similarity to the diatom-derived sphingolipid Δ10-desaturase^19^, and other olefin modifying oxygenases, hydrogenases and hydroxylases. Three conserved histidine cluster motifs (His boxes) are present in FGSG_09845 (aa 280–284, His box I; 317–321, His box II; 501–505, His box III) (Fig. 1B). The protein encoded by FGSG_09845 was predicted using TMHMM (http://www.cbs.dtu.dk/services/TMHMM/) to have five transmembrane helices, consistent with predicted topology for other membrane-bound desaturases. Therefore, it is proposed that ORF FGSG_09845 encodes the new desaturase, referred to as Δ10(E)-sphingolipid desaturase (Δ10(E)-SD).

### Knockout of Δ10(E)-SD gene abolishes fusaruside production

**Figure 2:**
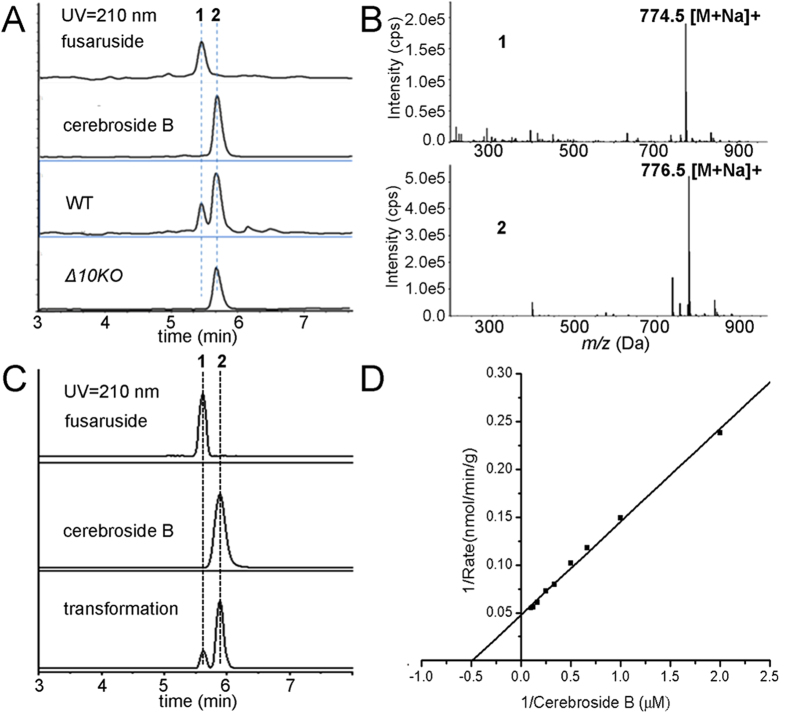
LC-MS screenings of sphingolipid-enriched fractions and *in vitro* enzymatic synthesis of fusaruside by recombinant Δ10(E)-SD. (**A**) LC-MS detection of **1** and **2** in sphingolipid-enriched fractions from WT and *Δ10KO* strains of *F. graminearum*. An EIC (extracted ion chromatogram) mode was used during LC-MS analysis. (**B**) MS spectra of **1** and **2**, corresponding to the Na^+^-liganded molecular ions at *m/z* 774.54907 (774.54905 calcd. for C_43_H_77_NO_9_Na) and 776.56470 (776.56477 calcd. for C_43_H_79_NO_9_Na). (**C**) Enzymatic production of **1** from **2**. Assay was performed with purified recombinant Δ10(E)-SD (100 μg) and **2** (1 μM) at 28 °C for 5 h. (**D**) Kinetic parameters for *in vitro* Δ10(E)-SD assay. Assays were accomplished with 100 μg enzyme and increasing amounts of **2** (0.5 to 10 μM), which were incubated at 28 °C for 20 min. Data are the mean of three replicates. The solid line represents a best fit analysis of the data, using the Lineweaver-Burk equation.

To ascertain whether the newly identified *F. graminearum* ORF encodes the sphingolipid 10,11-desaturase, the phenotypes of wild type (WT) and Δ10(E)-SD gene knockout (*Δ10KO*) strains of *F. graminearum* were compared. A vector containing 5’, 3’-sequences flanking the ORF of Δ10(E)-SD and selection marker of the gene encoding hygromycin B phosphotransferase was constructed and transformed into the *F. graminearum* (WT) protoplast cells by homologous recombination to form the mutant (*Δ10KO*) strain deprived of a functional allele of Δ10(E)-SD (Fig. S1). A sphingolipid-enriched fraction was prepared from WT and *Δ10KO* strains followed by LC-MS analysis. As illustrated in Fig. 2A, the wild type strain produced **1** whereas the *Δ10KO* strain did not, but both generated **2**. This suggests that Δ10(E)-SD is a sphingolipid desaturase catalyzing the *in vivo* generation of **1** from **2**.

### Generation of 1 from 2 under the catalysis of recombinant Δ10(E)-SD

**Figure 3:**
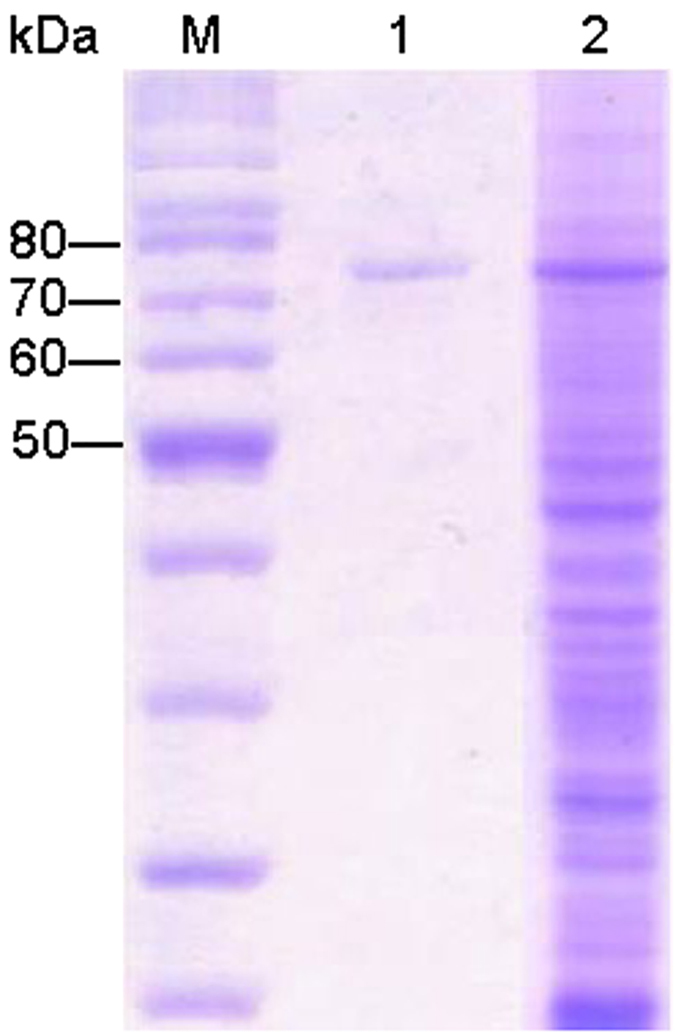
SDS-PAGE analysis of recombinant Δ10(E)-SD with an expected molecular weight of 70 kDa. Lane M, molecular mass markers; lane 1, fraction after purification by Ni-NTA affinity chromatography; lane 2, whole cell lysate of *P. pastoris* after methanol induction.

**Figure 4:**
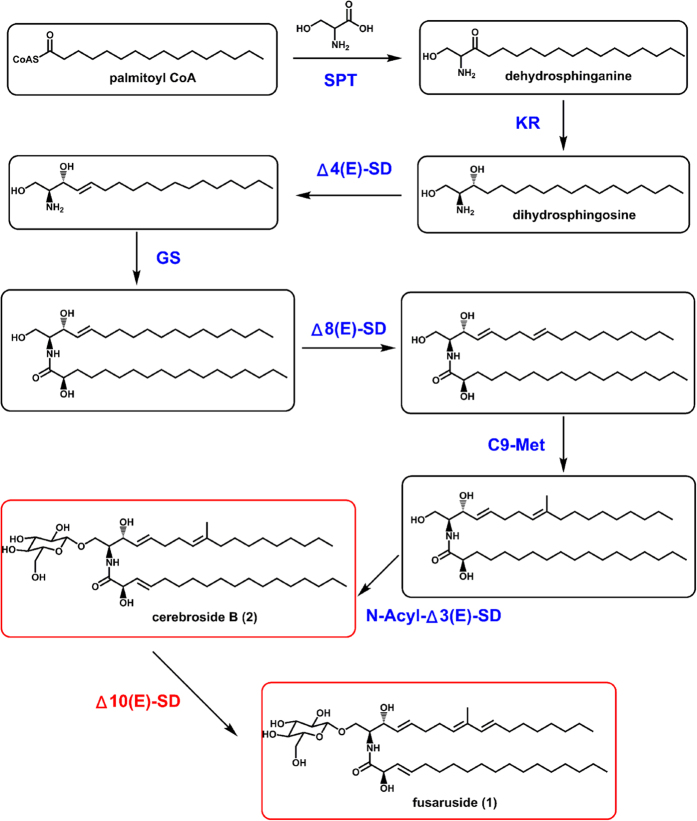
The fusaruside (**1**) biosynthetic pathway. SPT, serine palmitoyl transferase^9^; KR, ketosphinganine reductase^9^; Δ4(E)-SD, Δ4(E)-sphingolipid desaturase^20^; Δ8(E)-SD, Δ8(E)-sphingolipid desaturase (This study); GS, glucosylceramide synthase^20^; Δ10(E)-SD, Δ10(E)-sphingolipid desaturase (This study); C9-Met, C9-methyltransferase^20^; N-Acyl-Δ3(E)-SD, N-Acyl-Δ3(E)-desaturase^20^.

To demonstrate the enzymatic function *in vitro*, recombinant full-length Δ10(E)-SD was obtained using *Pichia pastoris* system. A crude fraction of recombinant Δ10(E)-SD carrying a hexa-histidine (6His)-epitope tag migrated on SDS-PAGE with a predicted molecular weight of 70 kDa, which was confirmed to be Δ10(E)-SD after purified by the Ni-NTA affinity chromatography (Fig. 3). To evaluate the enzymatic activity of homogeneous recombinant Δ10(E)-SD, purified enzyme (100 μg) was incubated with **2** (10 μM, dissolved in 1 mM CHAPS) in 1 mL buffer (pH 8.0, 2 mM NADH, 20 mM bicine, 50 mM NaCl, and 50 mM sucrose) for 5 h at 28 °C with gentle shaking, and the reactant mixture was analyzed by LC-MS (Fig. 2C). This *in vitro* reaction is comparable to the co-production of **1** and **2** by the endophytic fungi *F. semitectum* and *F. oxysporum*^11^,^12^. The steady-state kinetics of the reaction catalyzed *in vitro* by Δ10(E)-SD were examined by measuring the rate of conversion of **2** to **1** in the presence of 100 μg/mL enzyme and increasing gradually the substrate concentrations (0→10 μM). The reaction displayed Michaelis−Menten kinetics (Fig. 2D), with K_m_ for the substrate **2** at 2.06 μM, and V_max_ at 21.3 nmol/min/g. The enzyme has no reactivity towards 2-methylbutene-bearing molecules including citral, isochromophilone VI, nerolidol, phytolorvitamin D1 and cerebroside B derived ceramide (Figs. S2−S7), indicating that Δ10(E)-SD has high specificity for its substrate **2**. These data demonstrated that Δ10(E)-SD catalyzes desaturation of **2** to generate **1** *in vitro*. Correlating with the previously established common steps of sphingolipid biosynthesis, the complete biosynthetic pathway of **1** has been deduced as illustrated in Fig. 4.

### Analysis of glycosphingolipid in *ΔFgmt2* mutant

To determine whether the presence of the C9-methyl group is essential for the Δ10-desaturase activity, *ΔFgmt2* mutant strain was used^22^. Its lipid fraction was extracted and compared with that for *F. graminearum* WT strain. As illustrated in Fig. S11, nonmethylated cerebroside B could be detected, but nonmethylated fusaruside could not be found, implying that the presence of the C9-methyl group is essential for the sphingolipid Δ10-desaturase activity.

### Key amino acid residues of Δ10(E)-SD

As described above, three highly conserved histidine-rich boxes generally exist in the catalytic pockets of membrane-bound desaturases, and play essential roles in the catalysis of the enzymes^23^,^24^. By sequence alignment with other reported desaturases (Fig. 1B), Δ10(E)-SD has seven conserved His, an Asp and a Val in its histidine boxes.

To clarify the function of these amino acids, each of the residues was replaced by aliphatic residues. When residues H280, H284, H317, H320, H321, H504 and H505 were separately replaced by Ala, all mutant proteins were soluble, however, the 10,11-desaturating activity of the mutants toward **2** was completely lost (Table 1). This result suggests that the conserved histidines in the catalytic domain of Δ10(E)-SD are important for the catalytic effect of the enzyme. When residue D281 was replaced by Ala, the activity of the mutant protein was almost abolished whereas the mutation of V502 showed little effect on the enzyme activity. Taken together, these results suggest that the histidines and aspartic acid are hot spots of Δ10(E)-SD.

### Δ10(E)-SD improves survival of fungal strains in chill and salinity

**Figure 5:**
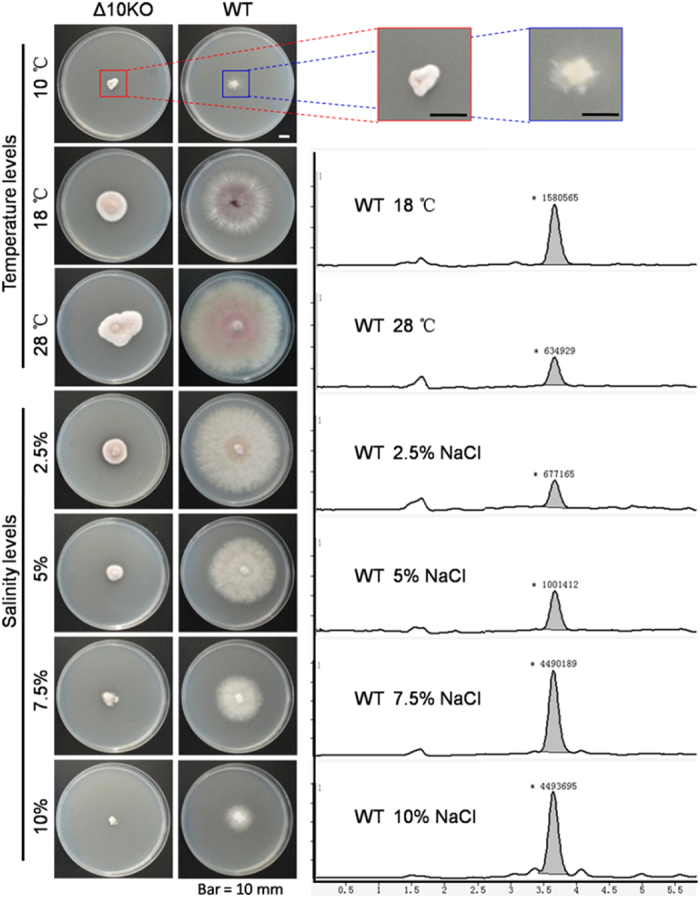
Survival of Δ10(E)-SD-expressing and *∆10KO* strains of *F. graminearum* at low temperature and high salinity. *Δ10KO* and wild type (WT) fungi were grown in PDA medium containing 0, 2.5, 5, 7.5, 10% NaCl and incubated for 6 days at 10, 18 and 28 °C. Compound **1** in fungal mycelia was quantified (right) by LC-MS run in EIC mode as detailed^28^. Cell growth at 10 °C was negligible with the collectable mycelium insufficient for analysis.

The fusaruside-generating endophytes *F. oxysporum* and *F. semitectum* IFB-121 reside in the perennial tree barks exposed inevitably to the repeated cold winter^11^,^12^. The gene encoding Δ10(E)-SD is absent in the genome of *F. oxysporum* CICC 41029, which does not produce **1**. *F. oxysporum* CICC 41029 used to harbour in tomato, an annual plant experiencing no winter. This collectively suggests the possibility that Δ10(E)-SD and/or its encoding gene may confer fungal resistance to low temperature. By extension, Δ10(E)-SD and/or its coding gene might also confer fungal resistance to salinity, since sphingolipids with 10,11-double bond at the trienic LCB motif as in case of **1** have been detected in some marine organisms^1^,^16^,^17^,^18^,^19^. The rationale for this idea is that sphingolipids may contribute to membrane integrity at low temperature^25^,^26^ and high osmotic pressure^27^. The hypothesis was tested by measuring growth and viability of WT and *Δ10KO* strains of *F. graminearum* challenged by low temperature or high salinity (Fig. 5). A sphingolipid-enriched fraction was also prepared from the strains, and analyzed by LC-MS to titrate **1** in mycelia according to the protocol detailed earlier^28^. The results showed that the stronger fungal resistance to cold and salinity correlated closely with the higher content of **1** in the mycelia (Fig. 5).

Similar data were obtained in the context of fungal cultures, where Δ10(E)-SD expressing *F. graminearum* CBS123657 and *F. semitectum* IFB-121 grew faster at low temperature or high salinity than did the Δ10(E)-SD deficient strains *F. oxysporum* CBS123668 and *F. verticillioides* CBS123670 (Fig. S8). Consistently, the enzyme activity was found to be 10.7 and 5.3 U/L at 18 and 28 °C, respectively, showing a potential enhancement of the enzyme at lower temperature. Similarly, the increment in NaCl concentration elevated the enzyme activity, which was found to be 7.1, 13.8 and 17.7 U/L at 2.5%, 5.0% and 7.5% NaCl, respectively (Fig. S9). For a robust confirmation, we have examined the fungal viability after frozen the strains for 72 hours at 0, −10, −20, −80 °C, indicating that fungal strains with Δ10(E)-SD are substantially more viable than the Δ10(E)-SD deficient ones (Fig. S10).

### Structural analysis of 1 and 2

The ^1^H NMR spectra of **1** and **2** (Figs. S12−S13) were identical to those of authentic materials^11^. In particular, five olefinic proton signals were exhibited at δ 5.83, 5.70, 5.56, 5.46, and 5.38 in the ^1^H NMR spectrum of **2**, suggesting the three double bonds in the molecule. In the ^1^H NMR spectrum of **1**, a total of four double bonds was required by the seven olefinic proton signals at δ 6.02, 5.81, 5.69, 5.59, 5.51, 5.41, and 5.33, and the splitting pattern of signals at δ 6.02 and 5.51 indicates the 10,11-double bond.

## Discussion

Selective desaturation of inert C-H bonds in aliphatic compounds is an important topic of chemistry^29^,^30^,^31^,^32^. Sphingolipids possess two aliphatic chains with different desaturation patterns, each resulting from a particular desaturase^9^,^20^. Compound **1** is a chemically rare and biologically promising fungal sphingolipid which is structurally unique in its 10,11-double bond at the sphingosine chain. The newly characterized Δ10(E)-SD (Fig. 1) catalyzes 10,11-desaturation of **2** to give **1**, representing a significant contribution of the study for the biotechnological supply of fusaruside as a selective immunosuppressive agent^13^,^14^. In compensation for the great difficulty in the selective chemical 10,11-desaturation of **2**, the present work describes an alternative and reliable supply of **1**; namely, genetic transformation of readily cultured fungal species (such as *Pichia pastoris*) with the ∆10(E)-SD gene, to confer the biosynthesis of **1** from **2** (more affordable^1^,^11^,^12^). The ∆10(E)-SD catalyzed transformation of **2** into **1** seems highly specific since the enzyme desaturated none of tested structurally related substrates (citral, isochromophilone VI, nerolidol, phytolorvitamin D1 and cerebroside B-derived ceramide) despite their possession of the 1,4-dialkylated 2-methylbutene scaffold (Figs. S2−S7). Thus, the regiospecific ∆10(E)-SD catalysis is mechanistically unique, which may require a combination of suitably spaced groups. Through genetic and/or enzymatic engineerings, ∆10(E)-SD could be a starter enzyme for the development of new biocatalyst(s) applicable for the selective desaturation of complex molecules.

∆10(E)-SD is a member of cytochrome b5 fusion proteins and belongs to the superfamily of membrane-bound desaturases. Previously, a cytochrome b5 fusion desaturase has been addressed to be responsible for the ∆10-desaturation of sphingolipid long chain bases in the marine diatom *Thalassiosira pseudonana*^19^. However, this plant ∆10-desaturase shares only 17.7% amino acid sequence similarity with ∆10(E)-SD from *F. graminearum*, indicating that sequence similarity is an unreliable factor in anticipating the catalyzing mechanism for members of the cytochrome b5 fusion desaturase gene family^33^, since the substrates for the two ∆10-desaturases are quite different. The presence of the C9-methyl group is essential for the activity of ∆10(E)-SD from *F. graminearum*, but not for the plant-derived sphingolipid Δ10-desaturase^19^. Along with the observation, the work confirms that cytochrome b5 fusion desaturases are quite diverse in terms of substrates and region-specificity mentioned elsewhere^34^.

Next, we succeeded in the heterologous expression of Δ10(E)-SD in *Pichia pastoris*, which was scaled up to a semi-preparative level. This facilitates both the enzymatic production of **1** from **2**, and further studies of the new desaturase having only 55.7% amino acid sequence identity with its closest counterpart Δ8(E)-SD (Fig. 1). Compound **2** is a common component of many fungi including certain edible mushrooms^1^. In view of the fact that sphingolipids are released by milk fermentation^35^,^36^, the heterologously expressible Δ10(E)-SD might find its potential application in modifying industrial strains in the milk industry, to produce fusaruside-containing milk products that may function against liver injury and colitis^13^,^14^.

Plant sphingolipid Δ8-unsaturation was found to improve low-temperature performance in *Arabidopsis*^37^. But no examination was performed concerning how the sphingolipid desaturation functions in microbes except for an observation that microbial survival and growth were determined by some small-molecule solutes such as trehalose, mannitol, arabitol, erythritol and glycerol^38^. This study demonstrates that Δ10(E)-SD confers upon fungal strains the ability to survive upon the exposure to low temperature and/or high salinity/high osmotic pressure (Fig. 5). These findings may/help explain unclarified observations such as: (i) the relationship between plasticity and sphingolipid unsaturation in eukaryotic cells^39^, (ii) the salinity-induced fungal synthesis of highly unsaturated sphingolipids^40^, (iii) the appropriateness of adopting unsaturated sphingolipids as molecular markers of fungal growth in cold sludge^41^, (iv) the accumulation of 10,11-unsaturated sphingolipids in some marine organisms^1^,^16^,^17^,^18^,^19^, (v) the host-dependent co-evolution of endophytes in species that thrive in harsh environments^42^,^43^, and (vi) the adaptation of some terrestrial microbes to the marine environment^44^,^45^.

Many *Fusarium* fungi are phytopathogenic, and their survival in the field in the cold winter may determine the prevalence of the *Fusarium* infection in the crops planted therein. We found that the *Fusarium* strains with Δ10(E)-SD gene have improved conidial survival rates (Fig. S10). Moreover, the interstrain genetic transformability highlights the possibility that Δ10(E)-SD may improve the survival of other microbes once they acquire the gene^46^. If so, Δ10(E)-SD could serve as an important target for conquering microbial pathogens with the gene, as implied by the enhancement of resveratrol chemotherapy by inhibiting the sphingolipid metabolism^47^.

In summary, the work characterizes Δ10(E)-SD as a new enzyme that catalyzes the last but key step in the fusaruside (**1**) biosynthesis. The amino acid sequence and substrate specificity of Δ10(E)-SD collectively distinguish it from its closest counterparts, Δ4(E)- and Δ8(E)-SDs as well as the C9-unmethylated Δ10-sphingolipid desaturase derived from a marine diatom^19^,^20^. This new desaturase has been heterogeneously expressed in *Pichia pastoris* GS115 with the recombinant enzyme functionally validated. The findings allow the desired large scale production of **1**, a pharmaceutically potential but supply-limited immunosuppressive agent. In addition to the improvement of fusaruside affordability, the new Δ10(E)-sphingolipid desaturase and its encoding gene function in the fungal adaptation to cold and high salinity. In aggregation, the investigation re-visualizes the significance of sphingolipid-related topics, and eases the follow-up investigations concerning the exact mechanism underlying both the fusaruside’s immunosuppression in mammals and the fungal resistance to cold and high salinity conferred by the new Δ10(E)-sphingolipid desaturase.

## Methods

### Reagents

Fusaruside (**1**) and cerebroside B (**2**) were extracted and purified in our lab. Citral, isochromophilone VI, nerolidol and phytolorvitamin D1 were purchased from Sigma Chemical Company (St. Louis, MO, USA). Trans Taq TM DNA polymerase and TransStart FastPfu DNA Polymerase were purchased from TransGen Biotech (Beijing, China). Nde I, Not I and other restriction enzymes were purchased from Takara (Japan). Plasmid Mini Kit I and Fungal DNA Kit were purchased from Omega (USA). RNAiso Plus was purchased from Takara (Japan). RevertAid First Strand cDNA Synthesis Kit was purchased from Thermo Scientific (USA). Ni-NTA agarose was purchased from Qiagen (Qiagen Co., Hilden, Germany).

### Bioinformatics

Using BLAST with Δ8(E)-SD (GenBank: KC787353) from *F. semitectum* IFB-121 as queries, the *Fusarium* comparative genome database was analysed. The resulting sequences were checked for the presence of histidine box motifs and analyzed using ClustalX (http://www.clustal.org/clustal2/).

### Strains and cultivation

*Fusarium semitectum* IFB-121 was isolated from the healthy bark of *Quercus variabilis*^11^. The genome-sequenced fungal strains *F. oxysporum* CICC 41029 (CBS 123668) isolated from tomato, *F. graminearum* 3.4598 (CBS 123657) from corn, and *F. verticillioides* (CBS 123670) from *Zea mays* were purchased from Centraalbureau voor Schimmelcultures (CBS) Fungal Biodiversity Centre in Netherlands. *Pichia pastoris* strain GS115 was obtained from Invitrogen, San Diego, USA. Fungi were grown on yeast extract-peptone-dextrose (YPD) agar containing in each liter yeast extract (10 g), peptone (20 g), dextrose (20 g), agar (20 g) and distilled water (1 liter). To screen for the fusaruside-producing strain, each of the genome-sequenced *Fusarium* species *F. oxysporum* CICC 41029, *F. graminearum* 3.4598 and *F. verticillioides* was grown simultaneously in 3 media including potato dextrose (PD: potato, 200 g; dextrose, 20 g; and distilled water, 1 liter), malt extract (ME: malt extract, 20 g; sucrose, 20 g; peptone, 1 g; and distilled water, 1 liter), and YPD media (free of agar), followed by incubation for 6 days at 28 °C with an agitation of 120 rpm.

### Sphingolipid isolation

Fresh mycelia (2–3 g) were suspended in H_2_O (5 mL) and held at 100 °C (in a boiling water bath) for 15 min. The cells were precipitated by centrifugation, and the lipid substance was extracted by shaking with 10 mL of chloroform/methanol (v/v, 1:1) overnight, followed by re-extraction with 9 mL of chloroform/methanol (v/v, 2:1) for at least 4 times. *F. graminearum* mycelia were homogenized in an Ultra-Turrax blender prior to the first extraction. Solvent removal from the filtrate under reduced pressure yielded a brown oily residue, which was dissolved in methanol with sonication, and filtered through a 0.45 μm membrane. The resulting sphingolipid enriched fraction was analyzed by LC-MS to validate the successful extraction of fungal sphingolipids.

LC-MS analyzes were performed on an Agilent 1200 series LC system coupled with an Agilent 6210 TOF mass spectrometer (Agilent Technologies, Palo Alto, CA, USA). For each analyte, five replicate MS measurements were conducted with LC separations performed on an Agilent ZORBAX Eclipse Plus C_18_ column (100 mm × 4.6 mm id, 3.5 μm; mobile phase: the methanol-water mixture at a flow rate of 0.4 μL/min for 10 min).

### Gene knockout

The genomic DNA of *F. graminearum* was extracted by Fungal DNA Kit (Omega, USA). The 708-bp fragment upstream of Δ10(E)-SD gene was amplified from the genomic DNA by PCR using 9845up-F/R as primers. The PCR product was isolated, digested with BglII/EcoRV, and inserted into the vector pSH75^48^ digested with the same restriction enzymes to give pDES1. The 836-bp fragment downstream of the Δ10(E)-SD gene was amplified using 9845down-F/R as primers, digested with BamHI/XbaI, and ligated into pDES1 to generate the disruption vector pDES2. Protoplasting and transformation of *F. graminearum* were carried out as described^49^. Transformants were overlaid with selective agar (100 μg/mL hygromycin B) at 28 °C for 7 days, and were transferred onto fresh CM plates containing 200 μg/mL hygromycin B. Correct integration of the knock-out cassette was confirmed by PCR using internal/external primers (Fig. S1).

### Cloning, expression and purification of Δ10(E)-SD

Total RNA of *F. graminearum* CBS123657 was extracted using RNAiso Plus (Takara, Japan), and total cDNA was synthesized by RevertAid First Strand cDNA Synthesis Kit (Thermo, USA). The gene encoding full-length Δ10(E)-SD was amplified from the total cDNA using 9845-F/R (Table S1). The gene was subcloned into vector pPICZa (Invitrogen) as an NdeI-NotI fragment and linearized with PmeI for transformation into *Pichia pastoris* GS115. Positive transformants (His^+^Mut^+^) were selected with Zeocin (200 μg/mL) and verified by gene sequencing. Pre-cultures of transformants (His^+^Mut^+^) were grown aerobically at 28 °C in buffered complex-glycerol medium (BMGY) in a shaking incubator (250~300 rpm). Cells were harvested and resuspended to OD_600_ = 1.0 with 250 mL buffered complex-methanol medium (BMMY) in **1**L flask. To induce protein expression under control of the AOX1 promoter, methanol was added to cultures at a final concentration of 0.5% every 12 h. After incubation at 28 °C for 60 h, yeast cells were collected, re-suspended in ice-cold lysis buffer (0.25 M sorbitol, 100 mM NaH_2_PO_4_/Na_2_HPO_4_, pH 7.4) and lysed by thorough sonication. The lysate was ultra-centrifuged (50,000 x g, 30 min, 4 °C), and the pellets were re-suspended in lysis buffer (contain 1% digitonin) with a further incubation for 1.5 h at 4 °C, followed by another centrifugation (24,000 × g, 30 min, 4 °C). The clean lysate was collected and purified by Ni-NTA affinity chromatography. The C-terminal His6-tagged recombinant Δ10(E)-SD was analyzed by SDS-PAGE.

### Protein analysis and enzyme assay

Protein was quantified by the method of Lowry^50^ with bovine serum albumin as standard. Enzyme assay was performed in 1 mL of 2 mM NADH, 20 mM bicine, 50 mM NaCl, 50 mM sucrose, pH 8.0 at 28 °C containing 100 μg Δ10(E)-SD and 10 μM **2** (dissolved in 1 mM CHAPS). The reaction was quenched with MeOH, and was monitored by LC-MS. To isolate the product, reactants were filtered, extracted with CHCl_3_/CH_3_OH (v/v, first 1:2, then 2:1) and purified by HPLC with CH_3_OH/H_2_O (v/v, 98:2). For steady-state kinetic analysis, 100 μg Δ10(E)-SD and 0.5−10 μM 2 were incubated in 1 mL reaction assay buffer (as above) at 28 °C for 20 min. Reactions were terminated with 75 μL MeOH, and the mixtures were analyzed by LC-MS. The kinetic parameters (K_m_ and V_max_) were determined using a Lineweaver-Burk plot.

### Site-directed mutagenesis

Site-directed mutagenesis of Δ10(E)-SD was performed using a typical overlap extension PCR strategy. The mutants were expressed in *Pichia pastoris* GS115, and the recombinant proteins were purified following the same protocol as the wild-type enzyme. The oligonucleotides used are shown in Table S1 with the underlined letters underscoring altered nucleotides.

### Substrate specificity of Δ10(E)-SD

A set of substrates (10 μM each) with a 1,4-dialkylated 2-methylbutene (including citral, isochromophilone VI, nerolidol, phytol, vitamin D1, and a ceramide derived from **2** through β-glucosidase catalyzed hydrolysis) was, after dissolved in 1 mM CHAPS, incubated separately with 100 μg Δ10(E)-SD in 1 mL buffer containing 2 mM NADH, 20 mM bicine, 50 mM NaCl, and 50 mM sucrose (pH 8.0) at 28 °C for 5 h. The reaction was quenched with MeOH, followed by LC-MS screenings.

### Activity of Δ10(E)-SD originated from fungal cells exposed to chill and salinity

To assess the relationship between enzyme activity and temperature, *F. graminearum* was cultivated at 200 rpm at 18 °C and 28 °C in YPD culture (5 g of yeast extract/L, 10 g of peptone/L, 10 g of D-glucose/L) for 5 days. To determine the dependence of enzyme activity on salinity, *F. graminearum* was cultivated at 200 rpm at 28 °C in YPD with NaCl at 0 to 7.5%. After cultivation, cells were collected by filtration and resuspended in ice-cold lysis buffer (0.25 M sorbitol, 100 mM NaH_2_PO4/Na_2_HPO_4_, pH 7.4) and lysed by thorough sonication. Enzyme assay was performed with 100 μL crude enzyme as in the experimental procedures, and one U is defined as the amount of crude enzyme that catalyzes the conversion of 10 μM fusaruside per minute at 28 °C.

### Fungal viability at low temperature

Conidia of fungi were suspended using 4.5 M glycerol solution. The spore suspensions were immediately exposed for 72 h to the low-temperature (0, −10, −20 and −80 °C)^38^. Upon completion of the treatment, conidia were immediately examined by light microscope (×100) to determine lethality^51^. One hundred conidia from each replicate spore suspension (i.e., 300 in total) were examined to determine percentage of survival; plotted values are the means of these independent triplicates, and bars show standard error of the mean.

### Isolation of 1 and 2

Both sphingolipids were isolated as described^11^. Briefly, lipid extraction was performed with CHCl_3_/CH_3_OH (v/v, first 1:2, then 2:1, each 24 h) twice at room temperature^52^. The total lipid extract (50−100 mg, dry weight) was fractionated by column chromatography on silica gel 60 (100−200 mesh, Merck)^53^, and eluted successively with 20 mL CHCl_3_/acetone (v/v, 4:1) and 40 mL acetone/CH_3_OH (v/v, 9:1). The second eluate was subjected to preparative TLC with acetone/toluene/water (91:30:8, v/v), to obtain the crude sphingolipid fraction, which was further purified by preparative HPLC (Hitachi semi-preparative column, 100% methanol; Hitachi, Tokyo, Japan) to yield **1** (*R*_*t*_ = 20.7 min) and **2** (*R*_*t*_ = 22.4 min). The ^1^H NMR spectra of **1** and **2** were acquired at 400 MHz on a Bruker Advance NMR spectrometer equipped with a 5 mm probe.

## Author Contributions

Y. T. and W. F. carried out the experiments. G. Y. Z. and Q. X. involved partially in the work. R. X. T. raised hypothesis, figured out strategy, designed experiments, supervised the project, and wrote the manuscript. All authors contributed to the data analysis and the manuscript preparation. Y. T., G. Y. Z. and W. F. contributed equally to the work.

## Additional Information

**How to cite this article**: Tian, Y. *et al.* Δ10(E)-Sphingolipid Desaturase Involved in Fusaruside Mycosynthesis and Stress Adaptation in Fusarium graminearum. *Sci. Rep.* **5**, 10486; doi: 10.1038/srep10486 (2015).

## Supplementary Material

Supplementary Information Supplementary Figures 1-6

## Figures and Tables

**Table 1:** The conversion rate of 2 into 1 by recombinant Δ10(E)-SD and its mutants.

**Mutation**	**conversion rate (%)**
WT	33
H280A	0
H284A	0
D281A	4
H317A	0
H320A	0
H321A	0
V502A	24
H504A	0
H505A	0

Assays were performed at 28 °C for 5 h with purified recombinant Δ10(E)-SD or its mutants (100 μg) and **2** (1 μM).
