# Supplementary material for: Δ10(E)-Sphingolipid Desaturase Involved in Fusaruside Mycosynthesis and Stress Adaptation in Fusarium graminearum
Source: Sci Rep. 2015 May 21;5:10486. doi: 10.1038/srep10486 (PMC4440215; doi:10.1038/srep10486)
Supplement: Supplementary Information — Supplementary Figures 1-6 [file srep10486-s1.pdf]

Supplementary material

Δ10(E)-Sphingolipid Desaturase Involved in Fusaruside Mycosynthesis and Stress Adaptation in *Fusarium graminearum*

Yuan Tian, Guo Y. Zhao, Wei Fang, Qiang Xu, and Ren X. Tan*

Institute of Functional Biomolecules, State Key Laboratory of Pharmaceutical Biotechnology, Nanjing University, Nanjing 210093, P. R. China

*To whom correspondence should be addressed. E-mail: rxtan@nju.edu.cn

Table S1.

Oligonucleotide primers used in this study (restriction enzyme sites are underlined, and "+" and "–" refer to the coding and non-coding strands, respectively). The mutated ORFs were confirmed by sequencing. The resulted plasmids were used for yeast transformation.

Name	primer Sequence (5′ → 3′)
D8-F	GAYCCKHGRTTCTTCAACATG
D8-R	DGTVCCDGTKCCRTGCATYTC
9845-F	GGAATTCCATATGATGGCGCATAGCTCTTTCGTT-3
9845-R	AAGGAAAAAAGCGGCCGCACTAGTGATGAGAGAGATCACCA
9845up-F	GAAGATCTGTGTAGTAGGAGTCAAAGC
9845up-R	TGCAGGATATCGGGGAGGAAGTCAAG
9845down-F	GCGGATCCCCCGAGAGCCTACGAAT
9845down-R	GCTCTAGAGCCTCAAAGAACAATACCA
external-F	GGCTGACCACCTGTTC
external-R	GCTCAATCGAAATATCACCTA
internal-F	CCAGGTGGTGATAAAGC
H280A-	CTATCGTTCACCGTTGCAGATGCTGGCCATATG
H280A+	CATATGGCCAGCATCTGCAACGGTGAACGATAG
D281A-	TCGTTCACCGTTCACGCAGCTGGCCATATGGGT
D281A+	ACCCATATGGCCAGCTGCGTGAACGGTGAACGA
H284A-	GTTCACGATGCTGGCGCAATGGGTATTACCCAC
H284A+	GTGGGTAATACCCATTGCGCCAGCATCGTGAAC
H317A-	TGGTGGAAGCGGAATGCAAACGTGCACCATGTC
H317A+	GACATGGTGCACGTTTGCATTCCGCTTCCACCA
H320A-	CGGAATCACAACGTGGCACATGTCGTCACCAAC
H320A+	GTTGGTGACGACATGTGCCACGTTGTGATTCCG
H321A-	AATCACAACGTGCACGCAGTCGTCACCAACGCT
H321A+	AGCGTTGGTGACGACTGCGTGCACGTTGTGATT
V502A-	GGCCTGCAGTTCCAAGCAATTCATCATCTCTTC
V502A+	GAAGAGATGATGAATTGCTTGGAACTGCAGGC C
H504A-	CAGTTCCAAGTTATTGCACATCTCTTCCCTCGT
H504A+	ACGAGGGAAGAGATGTGCAATAACTTGGAACTG
H505A-	TTCCAAGTTATTCATGCACTCTTCCCTCGTGTA
H505A+	TACACGAGGGAAGAGTGCATGAATAACTTGGAA

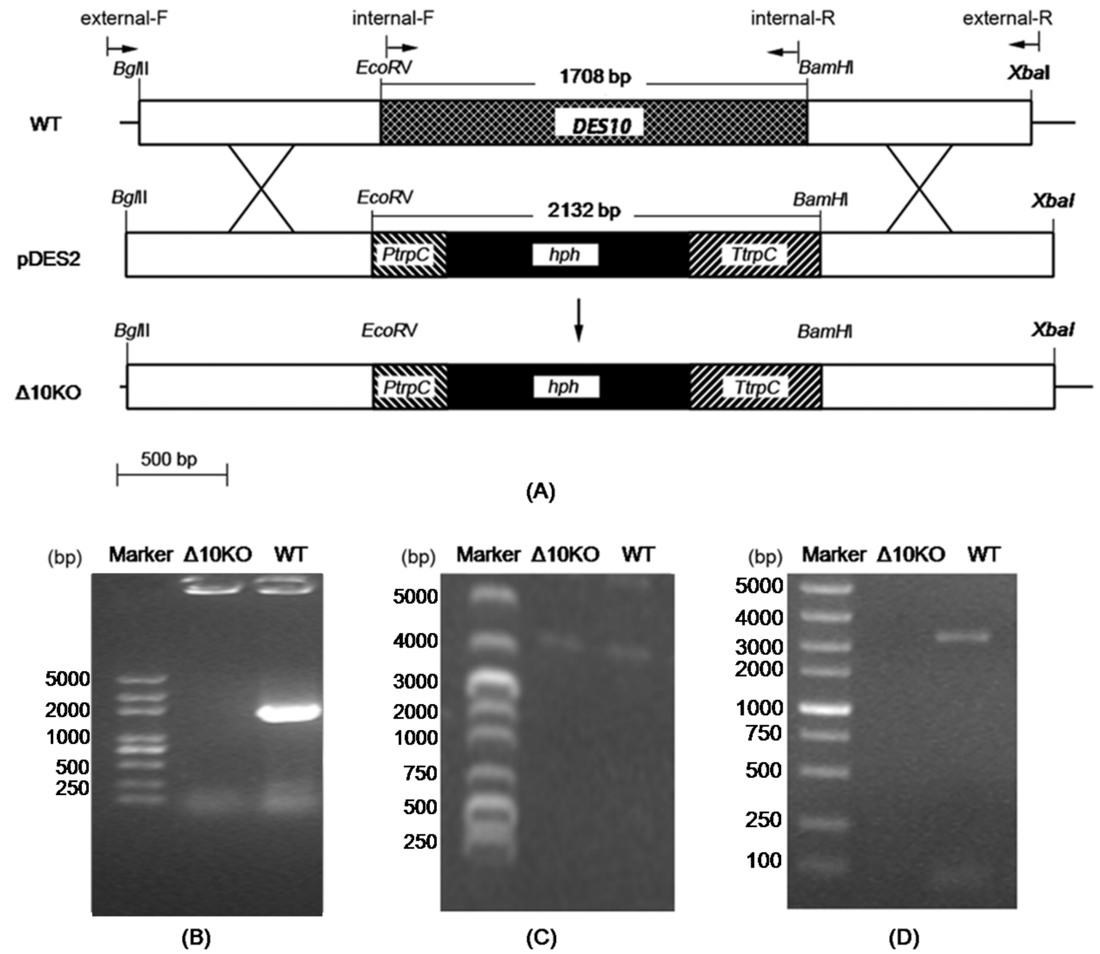

Fig. S1. Deletion of Δ10(E)-SD. (A**)** The disruption of Δ10(E)-SD gene by transforming DNA carrying the disruption cassette (pDES2) into wild-type (WT) chromosome to acquire the Δ10(E)-desaturase deleted (*Δ10KO*) chromosome. A depiction of the gene locus following a double cross-over with the disruption cassette containing *A. nidulans trpC* promoter (*PtrpC*), terminator (*TtrpC*) and hygromycin gene (*hph*) was designated, and positions of the restriction enzymes and primers were presented in the diagram. Diagnostic PCR experiments of WT and mutant (*Δ10KO*) strains were accomplished using (B) internal-F/R, (C) external-F/R, (D) external-F and internal-R as primers, respectively.

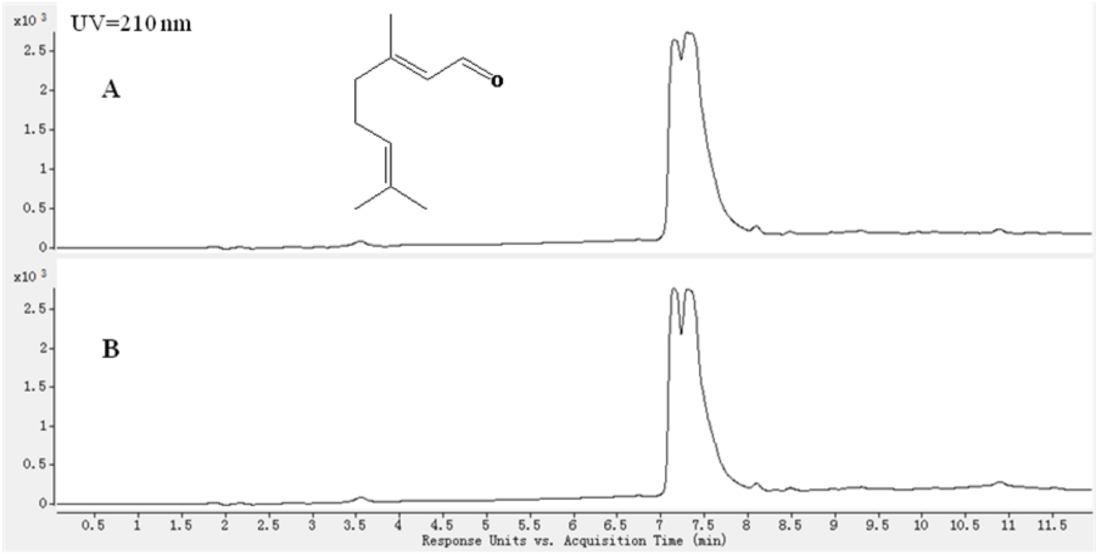

Fig. S2. Identical LC-MS profiles of citral before (**A**, authentic material) and after (**B**) treatment with recombinant Δ10(E)-SD. Assay was performed with 10 μM citral and 100 μg Δ10(E)-SD in 1 mL buffer containing 2 mM NADH, 20 mM bicine, 50 mM NaCl, and 50 mM sucrose (pH 8.0) at 28 ^{o}C for 5 h.

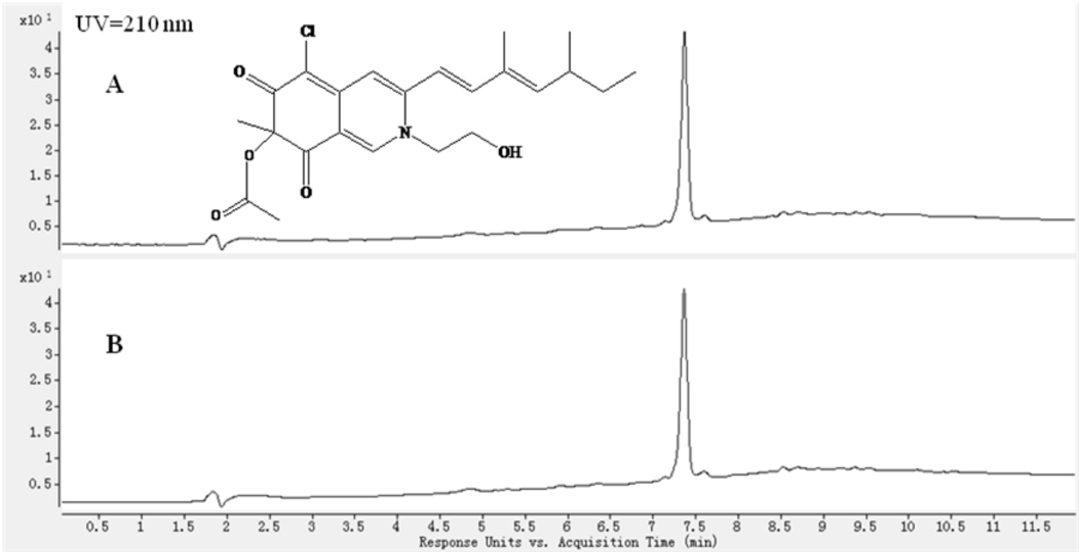

Fig. S3. Identical LC-MS profiles of isochromophilone VI before (**A**, authentic material) and after (**B**) treatment with recombinant Δ10(E)-SD. Assay was performed at 28 ^{o}C for 5 h with 10 μM isochromophilone VI and 100 μg Δ10(E)-SD in 1 mL buffer containing 2 mM NADH, 20 mM bicine, 50 mM NaCl, and 50 mM sucrose (pH 8.0).

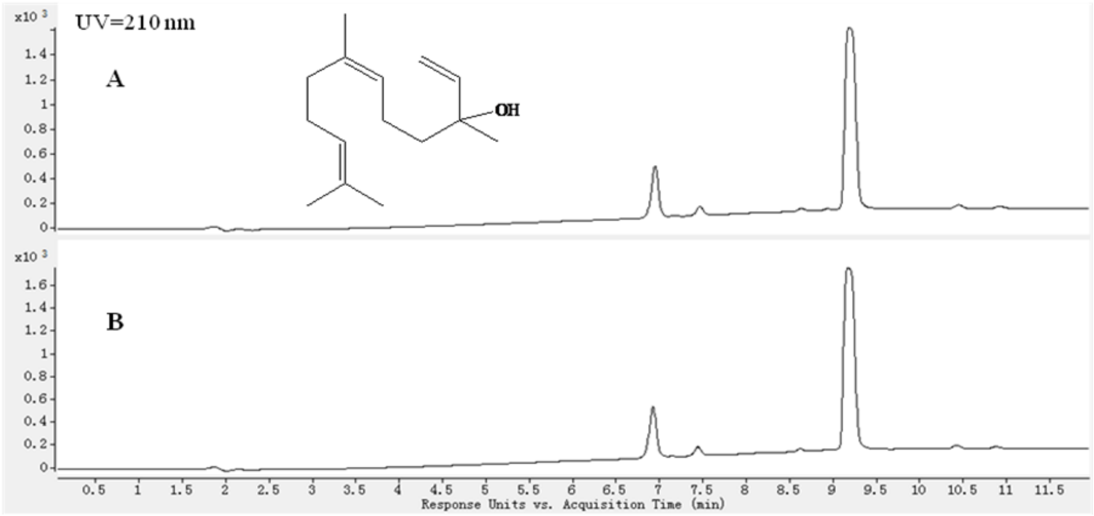

Fig. S4. Identical LC-MS profiles of nerolidol before (**A**, authentic material) and after (**B**) treatment with recombinant Δ10(E)-SD. Assay was performed at 28 ^{o}C for 5 h with 10 μM nerolidol and 100 μg Δ10(E)-SD in 1 mL buffer containing 2 mM NADH, 20 mM bicine, 50 mM NaCl, and 50 mM sucrose (pH 8.0).

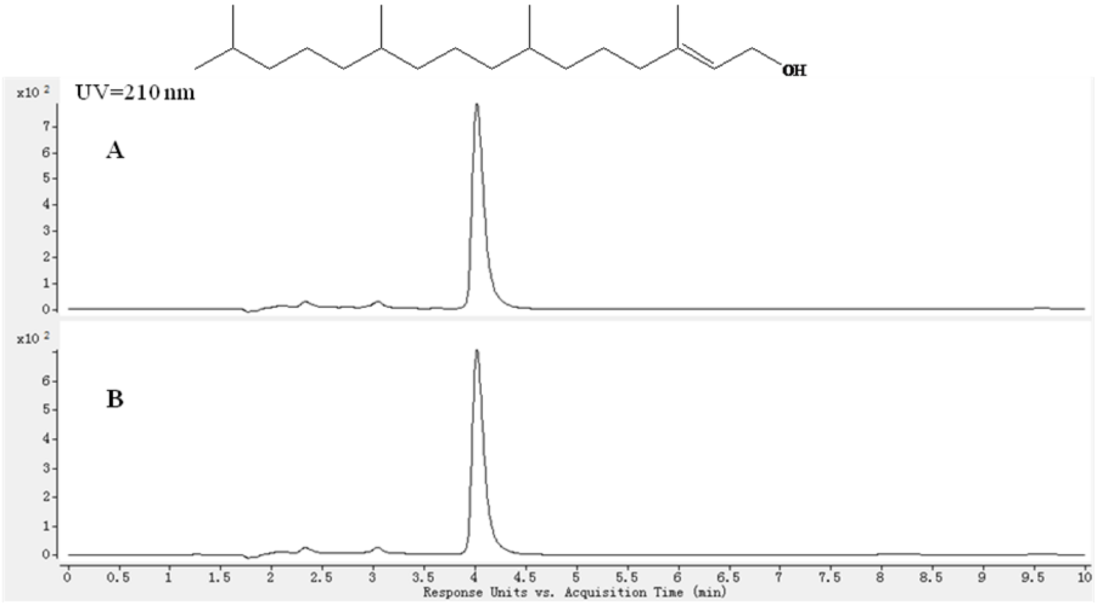

Fig. S5. Identical LC-MS profiles of phytol before (**A**, authentic material) and after (**B**) treatment with recombinant Δ10(E)-SD. Assay was performed at 28 ^{o}C for 5 h with 10 μM phytol and 100 μg Δ10(E)-SD in 1 mL buffer containing 2 mM NADH, 20 mM bicine, 50 mM NaCl, and 50 mM sucrose (pH 8.0).

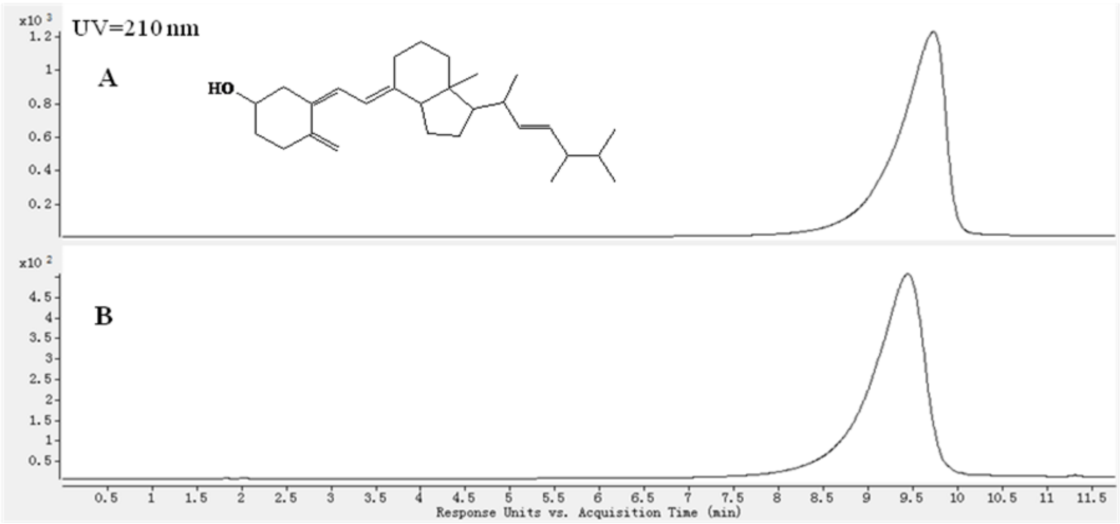

Fig. S6. Identical LC-MS profiles of vitamin D1 before (**A**, authentic material) and after (**B**) treatment with recombinant Δ10(E)-SD. Assay was performed at 28 ^{o}C for 5 h with 10 µM vitamin D1 and 100 µg Δ10(E)-SD in 1 mL buffer containing 2 mM NADH, 20 mM bicine, 50 mM NaCl, and 50 mM sucrose (pH 8.0).

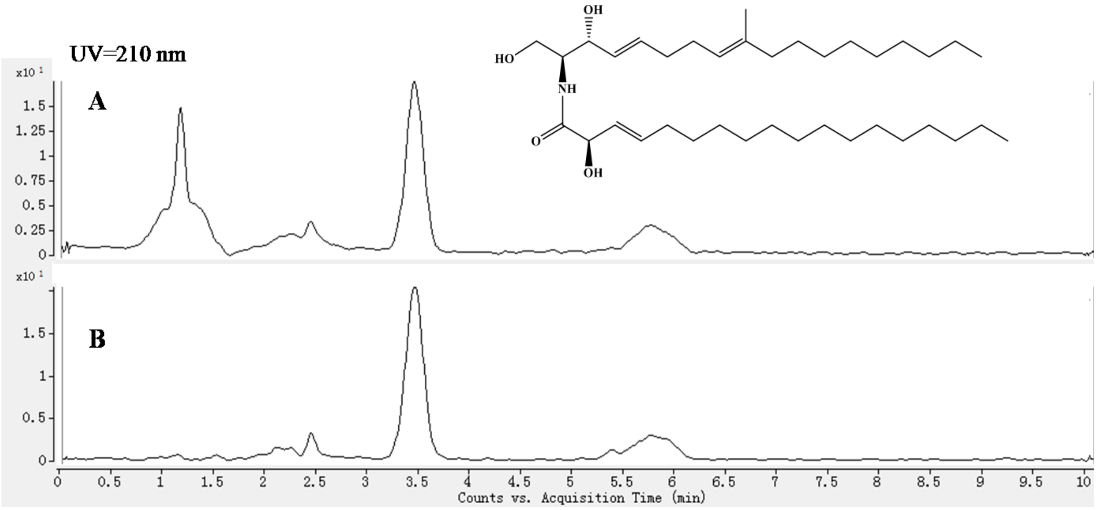

Fig. S7. Identical LC-MS profiles of cerebroside B-derived ceramide before (A, authentic material) and after (B) treatment with recombinant Δ10(E)-SD. Assay was performed at 28 ^{o}C for 5 h with 10 µM ceramide and 100 µg Δ10(E)-SD in 1 mL buffer containing 2 mM NADH, 20 mM bicine, 50 mM NaCl, and 50 mM sucrose (pH 8.0).

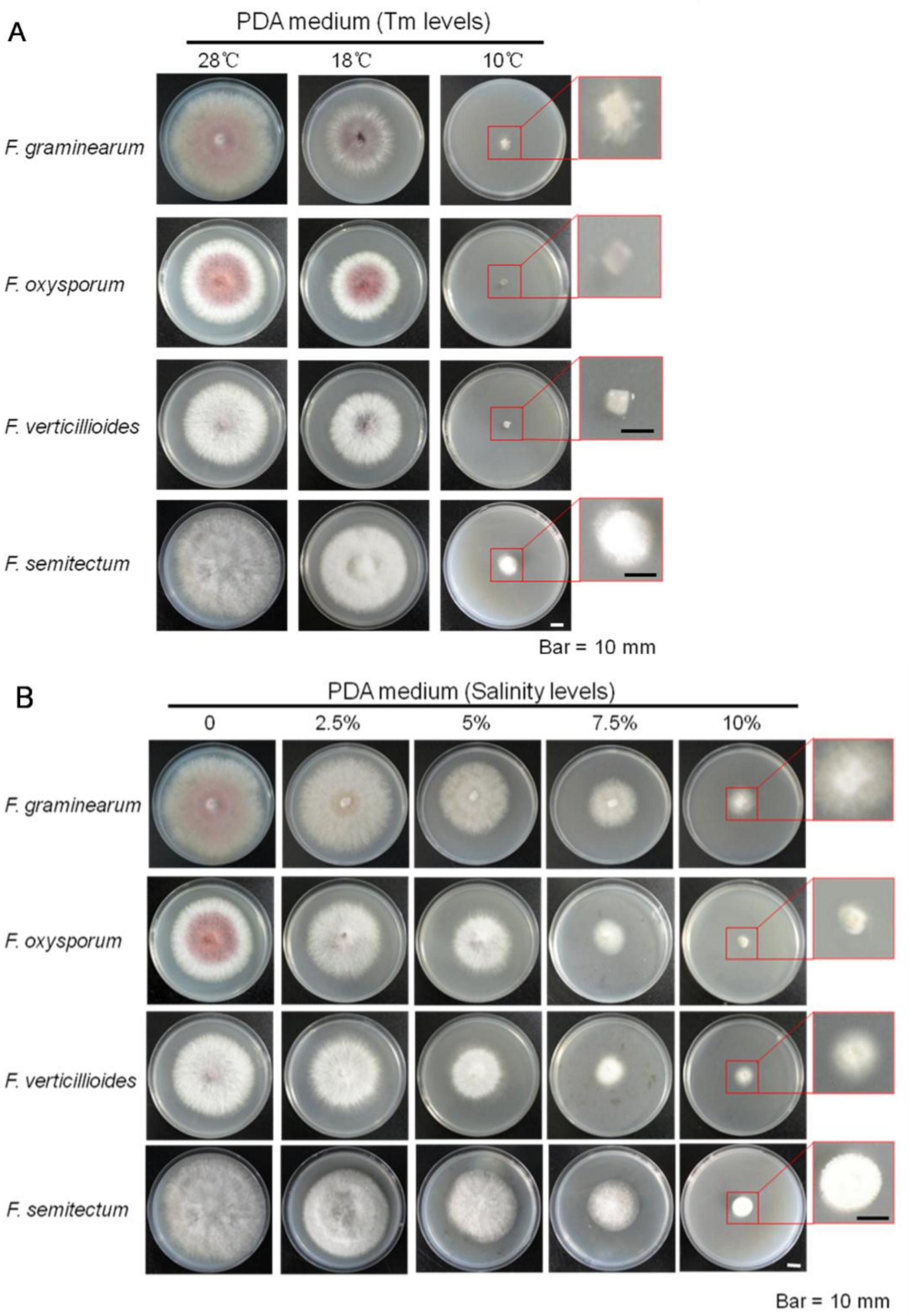

Fig. S8. Interspecies growth comparison. The growth rate of Δ10(E)-SD gene-having *F. graminearum* CBS123657 and *F. semitectum* IFB-121 was faster than that of Δ10(E)-SD gene-deficient *F. oxysporum* CBS123668 and *F. verticillioides* CBS123670, when challenged equally by low temperature (A) and high salinity (B). The mycelium was placed in the centre of the PDA medium (containing 0, 2.5, 5, 7.5, 10% NaCl, respectively) plates and incubated for 6 days at 10, 18 and 28℃.

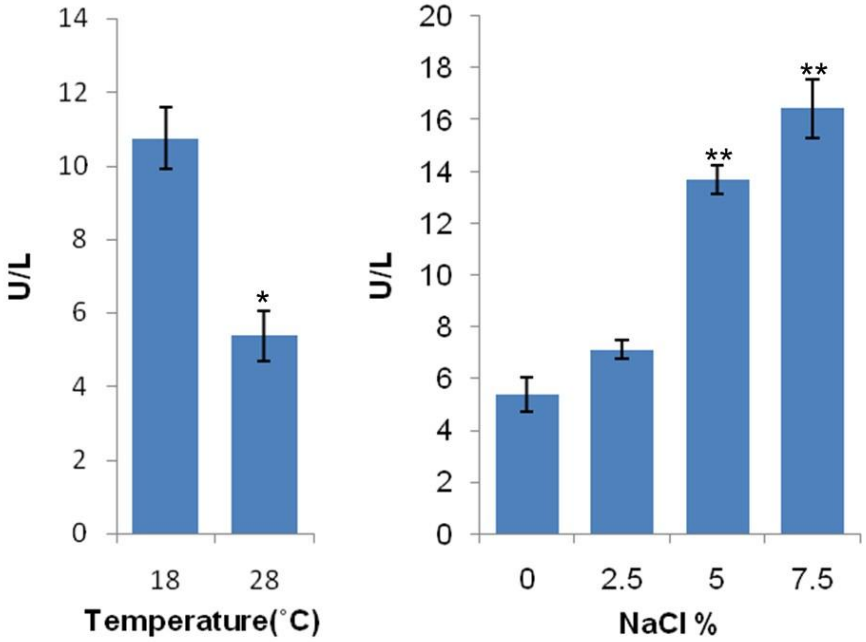

Fig. S9. Activity comparison of Δ10(E)-SD derived from fungal cells grown with exposure to chill and salinity. *P<0.05, **P<0.01 Student t test.

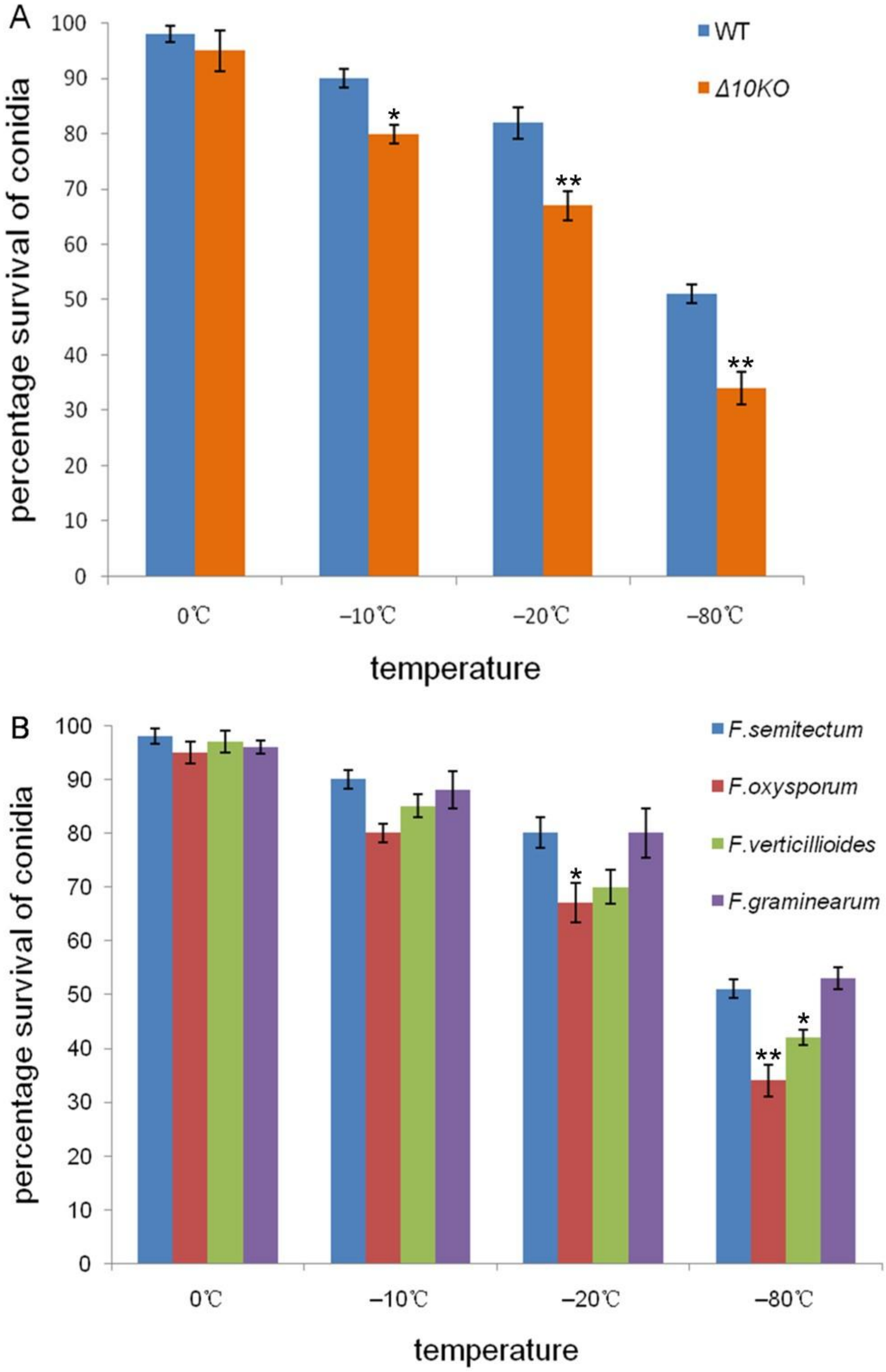

Fig. S10. Comparison for conidial survival rates of the fungal strains with and without the Δ10(E)-SD encoding gene after a 72-hour exposure to low temperature. (A) The spore viability of the WT type strain of *F. graminearum* is higher than that of the *Δ10KO* strain. (B) The spore viability of Δ10(E)-SD producing *F. graminearum* CBS123657 and *F. semitectum* IFB-121 was higher than that of Δ10(E)-SD deficient *F. oxysporum* CBS123668 and *F. verticillioides* CBS123670. *P<0.05, **P<0.01 Student t test.

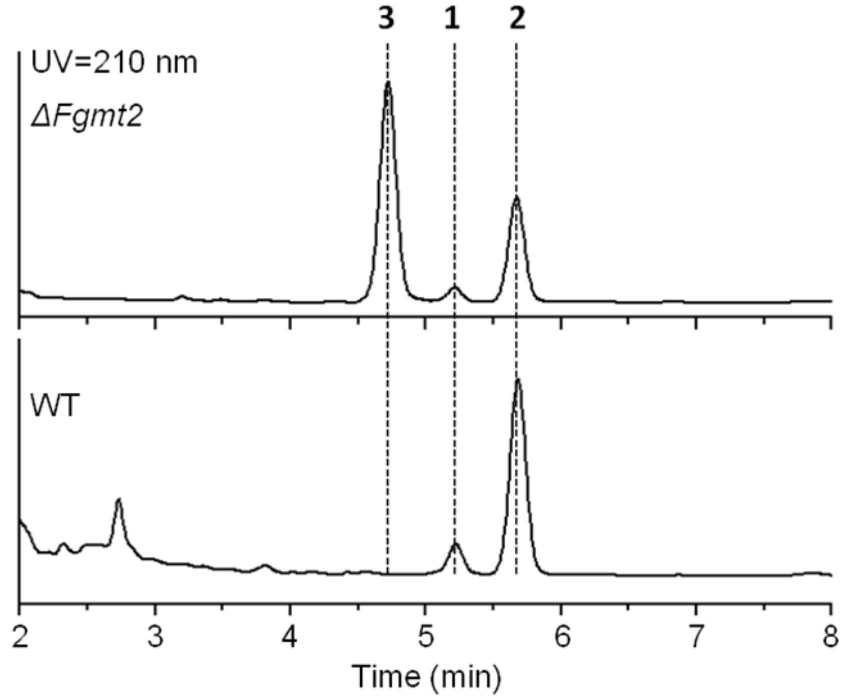

Fig. S11. Analysis of lipids isolated from the *ΔFgmt2* mutants and wild-type strain of *F. graminearum*. **1**: fusaruside, **2**: cerebroside B**, 3**: nonmethylated cerebroside B.

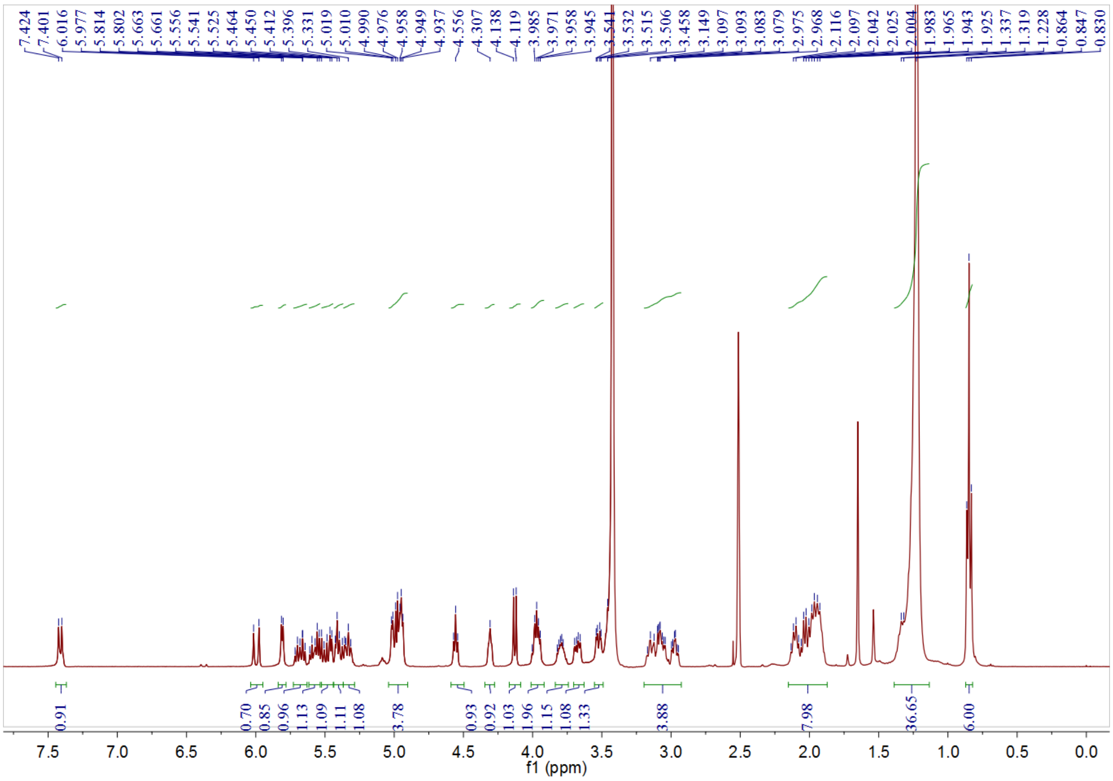

Fig. S12. ^{1}H NMR of fusaruside (**1**) in DMSO-d_6 (400 MHz).

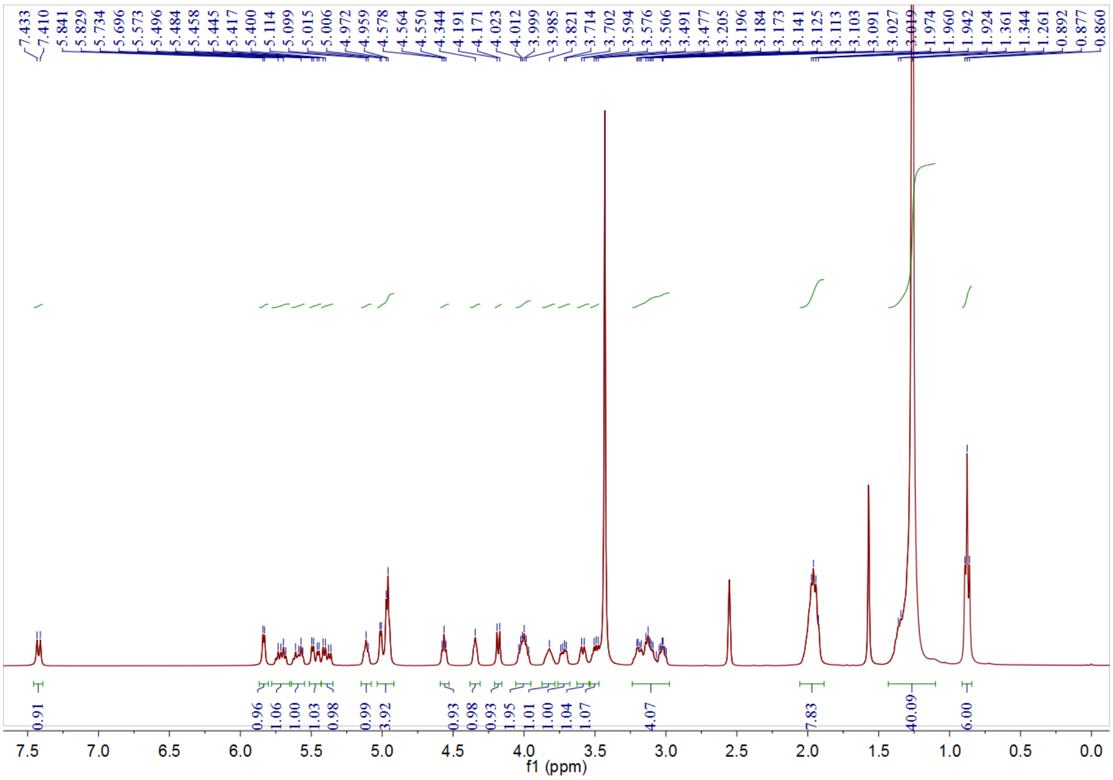

Fig. S13. ^{1}H NMR of cerebroside B (**2**) in DMSO-d_6 (400 MHz).
